# Unusual Presentation of West Nile Virus Meningitis With Cerebrospinal Fluid (CSF) Neutrophilia in a 68-Year-Old Male

**DOI:** 10.7759/cureus.85520

**Published:** 2025-06-07

**Authors:** Rawad Daniel Arja, Jonathan Kuo, Dima Youssef

**Affiliations:** 1 Internal Medicine, Valley Health System, Las Vegas, USA; 2 Internal Medicine, College of Osteopathic Medicine, Touro University Nevada, Henderson, USA

**Keywords:** cerebrospinal fluid (csf), headache, neuroinvasive west nile virus, neutrophilia, viral meningitis

## Abstract

West Nile virus (WNV), a mosquito-borne flavivirus, can cause neuroinvasive disease, including meningitis, encephalitis, and acute flaccid paralysis. WNV meningitis typically presents with lymphocytosis in the cerebrospinal fluid (CSF). We report a case of a 68-year-old male who presented with dizziness, syncope, and head trauma, accompanied by headache, neck pain, and fever (102.3 °F), but without confusion or photophobia. The initial workup was unremarkable, but the lumbar puncture revealed elevated cerebrospinal fluid (CSF) opening pressure, neutrophilic pleocytosis, and increased red blood cells, protein, and glucose levels. CSF serology was positive for WNV IgM, and other infectious etiologies were excluded. This case illustrates that WNV meningitis may present with atypical CSF neutrophilic predominance, emphasizing the need to consider WNV in the differential diagnosis of meningitis, especially during mosquito season or in endemic areas, even when CSF findings are non-classical. Early recognition and diagnostic testing are essential for appropriate management.

## Introduction

West Nile virus (WNV) is an arbovirus transmitted primarily by the Culex tarsalis mosquito, causing a wide range of clinical manifestations, from mild febrile illness to severe neuroinvasive diseases such as meningitis, encephalitis, and acute flaccid paralysis [1]. Since its emergence in the United States in 1999, WNV has become the leading cause of mosquito-borne disease in the country, now reported in all 50 states as of 2025, with a significant number of annual cases. 

Classically, WNV meningitis or encephalitis presents with lymphocytosis in the cerebrospinal fluid (CSF). However, emerging evidence, which will be discussed in our paper, suggests that neutrophilia may be an early and prominent finding, particularly in immunocompromised patients.

## Case presentation

A 68-year-old male with a medical history of hypertension, type 2 diabetes mellitus, and chronic low back pain presented following a syncopal episode associated with dizziness, lightheadedness, and minor head trauma. He reported persistent daily frontal headaches and neck pain but denied confusion, photophobia, nausea, or vomiting. Initial vitals were notable for a fever of 102.3 °F, elevated blood pressure, and tachypnea, with normal oxygen saturation. Physical examination revealed no focal neurological deficits and intact cranial nerves. The persistent daily frontal headaches and neck pain gradually improved before discharge.

A non-contrast computed tomography scan of the head and cervical spine demonstrated no acute intracranial abnormalities but showed moderate central spinal stenosis at the C4-C5 level, with severe right-sided neural foraminal stenosis at C5 and C6. Brain magnetic resonance imaging and magnetic resonance angiography were unremarkable. Carotid Doppler ultrasound identified bilateral atherosclerotic plaques with elevated flow velocity in the right internal carotid artery, but it does not explain the patient's headache or fever. Chest X-ray and electrocardiogram were also unremarkable. 

The patient’s labs remained normal throughout the stay, except for the CSF findings. Lumbar puncture revealed elevated CSF opening pressure. CSF analysis showed an elevated white blood cell count with neutrophilic predominance, increased red blood cells, elevated total protein, and mildly elevated glucose levels (Table 1). CSF polymerase chain reaction (PCR) testing and cultures for common bacterial and viral pathogens were negative. However, CSF serology later returned positive for WNV IgM antibodies, confirming the diagnosis of acute WNV neuroinvasive disease. 

**Table 1:** Comparison of CSF findings. CSF, cerebrospinal fluid; HSV, herpes simplex virus; CMV, cytomegalovirus; VZV, varicella-zoster virus

Parameter	Patient’s CSF	Normal CSF	Expected in WNV
Opening pressure	22 cm H_2_O	10-20 cm H_2_O	Usually normal or mildly elevated
WBC	37 cells/µL	0-5 cells/µL	10-100 cells/µL
Neutrophils	22 cells/µL (59%)	0 cells/µL (0%)	Predominantly lymphocytes, <20% neutrophils
RBC	276 cells/µL	0 cells/µL	0 cells/µL
Total protein	37 mg/dL	15-45 mg/dL	50-150 mg/dL
Glucose	72 mg/dL	40-70 mg/dL	Normal
West Nile IgM	Positive	Negative	Positive
Listeria monocytogenes	Negative	Negative	Negative
Streptococcus pneumoniae	Negative	Negative	Negative
Neisseria meningitidis	Negative	Negative	Negative
HSV	Negative	Negative	Negative
CMV	Negative	Negative	Negative
VZV	Negative	Negative	Negative
Enterovirus	Negative	Negative	Negative

Before diagnosis, the patient was started on empiric antibiotics with vancomycin, ceftriaxone, and ampicillin for presumed bacterial meningitis. Supportive care with antipyretics, analgesics, and physical therapy was also provided. After a seven-day course of antibiotics, ongoing symptoms and positive WNV serology supported a viral etiology. The patient gradually improved and was discharged with instructions to follow up with an infectious disease specialist. 

## Discussion

Lymphocytosis is considered the classic CSF finding in WNV neuroinvasive disease. However, recent reports have highlighted examples of CSF neutrophil predominance, particularly in the early disease course. We conducted a literature search with the PubMed database with keywords “West Nile Virus,” “Neutrophil,” and “Cerebrospinal Fluid,” identifying 15 results. Of these, six studies demonstrated neutrophil-predominant CSF patterns (Table 2).

Previous reports have shown that in early-onset WNV with primarily IgM antibodies, CSF neutrophil predominance is seen in 45% of meningitis patients and 37% of encephalitis patients [2-4]. However, many of these patients experienced a shift from neutrophil to lymphocyte predominance later in their disease course [2]. In this case, the patient remained neutrophil predominant. Other reports reveal neutrophilia in either immunocompromised or paralyzed patients, emphasizing a susceptible population [5-7]. 

This case adds to the growing body of evidence revealing that WNV meningitis can present similarly to bacterial meningitis with neutrophilic CSF pleocytosis. This finding may cause delays in diagnosis or lead to mismanagement, particularly in non-endemic regions. Clinicians should maintain a high index of suspicion for WNV, especially during peak mosquito season and in cases of CSF neutrophilia, to ensure timely diagnosis and appropriate care.

**Table 2:** Overview of neutrophilia in WNV meningitis patients: a review of case studies and clinical reports. This table provides a review of various case studies on WNV neuroinvasive disease, focusing on CSF findings. It summarizes key cases that highlight the presence of neutrophilia in the early stages of infection and the eventual shift to lymphocytic predominance, reflecting the variability in immune responses across different patient populations. WNV, West Nile virus; CSF, cerebrospinal fluid

	Authors	Key findings	Special with this case
Case 1 (2021)	Falcinella et al. [2]	Describes three cases of WNV neuroinvasive disease with initial CSF neutrophilia, followed by a shift to lymphocytic predominance	Eventual shift to lymphocyte predominance
Case 2 (2006)	Tyler et al. [3]	Study of 250 WNV patients with meningitis or encephalitis, where 45% of meningitis and 37% of encephalitis patients had a minimum of 50% neutrophils in their CSF samples	These patients had early presentations of WNV with IgM.
Case 3 (2004)	Crichlow et al. [4]	Describes seven patients with WNV with CSF neutrophilic pleocytosis rather than typical lymphocytic pleocytosis	These patients had early presentations of WNV with IgM.
Case 4 (2021)	Kasule et al. [5]	Twenty-four solid organ transplant recipients with WNV, initial CSF with neutrophil predominance	These patients were immunocompromised.
Case 5 (2005)	Sejvar et al. [6]	Early CSF findings in WNV show neutrophilic pleocytosis, especially in patients with paralysis.	These patients were paralyzed.
Case 6 (2010)	Bai et al. [7]	Highlights the presence of neutrophilia in WNV, emphasizing variability in immune responses	Emphasis on immune response in mice

## Conclusions

This case highlights an atypical presentation of WNV meningitis, marked by CSF neutrophilic predominance rather than the expected lymphocytosis. Such findings mimic bacterial meningitis and complicate timely diagnosis, potentially leading to unnecessary antibiotic use. Clinicians should remain vigilant for WNV as a possible etiology of meningitis during mosquito season or in endemic regions, even when CSF profiles are non-classical. Early consideration of WNV in the differential diagnosis and prompt serologic testing are essential to guide appropriate management and avoid diagnostic delays. 
